# Investigating generative AI models and detection techniques: impacts of tokenization and dataset size on identification of AI-generated text

**DOI:** 10.3389/frai.2024.1469197

**Published:** 2024-11-19

**Authors:** Haowei Hua, Co-Jiayu Yao

**Affiliations:** ^1^The Culver Academies, Culver, IN, United States; ^2^Anhui Polytechnic University, Wuhu, China

**Keywords:** generative artificial intelligence (GenAI), machine learning, natural language processing, writing assessment, ChatGPT, Claude, text classification

## Abstract

Generative AI models, including ChatGPT, Gemini, and Claude, are increasingly significant in enhancing K–12 education, offering support across various disciplines. These models provide sample answers for humanities prompts, solve mathematical equations, and brainstorm novel ideas. Despite their educational value, ethical concerns have emerged regarding their potential to mislead students into copying answers directly from AI when completing assignments, assessments, or research papers. Current detectors, such as GPT-Zero, struggle to identify modified AI-generated texts and show reduced reliability for English as a Second Language learners. This study investigates detection of academic cheating by use of generative AI in high-stakes writing assessments. Classical machine learning models, including logistic regression, XGBoost, and support vector machine, are used to distinguish between AI-generated and student-written essays. Additionally, large language models including BERT, RoBERTa, and Electra are examined and compared to traditional machine learning models. The analysis focuses on prompt 1 from the ASAP Kaggle competition. To evaluate the effectiveness of various detection methods and generative AI models, we include ChatGPT, Claude, and Gemini in their base, pro, and latest versions. Furthermore, we examine the impact of paraphrasing tools such as GPT-Humanizer and QuillBot and introduce a new method of using synonym information to detect humanized AI texts. Additionally, the relationship between dataset size and model performance is explored to inform data collection in future research.

## 1 Introduction

Artificial intelligence (AI) is an increasing powerful tool used in multiple fields, including education. AI and machine learning (ML) can handle large datasets, perform complex computations, and automate repetitive tasks with high precision. The automation process reduces operational costs and enables predictive modeling (Das et al., 2015). For fraud detection in the financial industry, AI technologies enhance financial institutions' ability to detect and prevent fraudulent activities with accuracy and efficiency. AI and ML algorithms are excellent at identifying unusual patterns of behavior in large datasets. Users are screened by analyzing their behavior across multiple channels and touch-points to identify anomalies. AI-powered systems also analyze transactions in real time, reducing the potential negative impact of fraud for account holders and the bank (Khanzode and Sarode, 2020). ML methods such as logistic regression, decision trees, and support vector machines (SVMs) with deep learning networks like convolutional neural networks (CNNs) are frequently used to detect fake news and adverts. Studies show the effectiveness of various AI techniques in combating the spread of fake news on social media platforms (Kaur et al., 2019). Similarly, for detecting fake reviews to ensure costumers receive accurate feedback on products, ML supervised learning algorithms such as random forests or natural language processing, including sentiment analysis and linguistic analysis, are useful tools. In particular, advanced ML models integrating behavioral and contextual features achieve higher detection rates than traditional methods (Mukherjee et al., 2013).

Generative AI such as Chat-GPT also present many challenges. For example, in education, Chat-GPT can generate answers or revise students' work to produce well-written essays in a short amount of time. Thus, although it reduces the resources and costs of creating academic assignments, there are ethical problems and serious concerns about uncontrolled AI usage in the academic field. Generative AI (GenAI) poses new challenges to academic integrity by enabling people to generate original-looking content that can be used to cheat on assignments, exams, and other assessments. Students using tools like Chat-GPT to write essays could bypass the traditional leaning process, undermining educational standards. Therefore, the development of GenAI introduces the need for robust policies, ethical guidelines, and detection tools to ensure its responsible usage (Akkaş et al., 2024). A comparison of essays generated by Chat-GPT with those written by humans, examining language mastery, complexity of vocabulary, and structural sophistication, found GPT-4 and GPT-3 generally scored higher than humans, suggesting a potential trend for students to use AI to cheat on writing assignments (Herbold et al., 2023). In English as a second language settings, the online tools currently available, such as GPT-Zero, offer inconsistent results, especially in non-English contexts, highlighting the need for more reliable tools. The lack of awareness of the accuracy of AI generated content also further validates the need for a better detection method (Alexander et al., 2023).

Various studies have investigated detection of machine generated texts in academic fields. SVMs, a type of traditional machine learning algorithm, have demonstrated 100% accuracy in identifying human-generated texts. Compared with other existing detection tools, they offer valuable insights for improving detection methods. Cingillioglu (2023) used an n-gram bag-of-words discrepancy language model as input for a classifier to detect AI-generated essays. To detect essays written by both native and non-native Arabic-speakers and AI-generated essays, a SVM-based classifier with term frequency-inverse document frequency (TF-IDF) outperformed other models with an accuracy score of 91.14% (El Kah et al., 2023). Another study used neural network-based and feature-based methods to detect AI-generated content. It focused on one-class learning models and evaluated the performance of existing AI-text detectors such as GPT-Zero and Turnitin. Overall, the fine-tuned large language model detected AI generated essays (created with GPT-3.0) with 99% accuracy (Yan et al., 2023). Corizzo and Leal-Arenas (2023) used a deep neural network architecture and different features including linguistic features and text modeling, creating a system that achieved 98% accuracy, testing English and Spanish essays written by L2 learners and essays generated using GPT-3.0. In deep learning models, the BERT model achieves 97.71% accuracy (Wang et al., 2024). The DeBERTa model, applied to English text, achieves a Macro F1 score of 57.1% (Morales-Márquez et al., 2023). In models for detecting AI generated text, certain attributes such as perplexity and semantic features are found to be effective for distinguishing between human-written and machine-generated texts (Mindner et al., 2023). Zeng et al. (2024) developed a two-step approach to identifying human-written and AI-generated content in hybrid essays that utilized encoder training and prototype-based boundary detection. The approach achieved approximately 75% accuracy in boundary detection. Another deep learning model integrating long short-term memory, transformers, and CNN scored 99.8% accuracy identifying AI-generated texts created using GPT-3.5 (Mo et al., 2024). Another approach to detecting AI text leverages the properties of the log probability function of large language models. A zero-shot detection model achieves a high accuracy of 99% for GPT-3 texts (Mitchell et al., 2023). Using a combination of academic judgement and AI detection software to identify machine-generated content created using GPT-4, the AI detection tool is able to identify 91% of the submissions but only 54.8 % of each text data's original content is classified as AI-generated (Perkins et al., 2024). In a general overview of AI detectors that are currently available online for free (Chaka, 2024), 30 AI detectors were compared for detecting AI content in written essays. Only a few AI detectors such as Copyleaks performed well when crossing English as first language(L1) and English as second language(L2) settings. The results indicate AI detectors' unreliability when determining the source of essays. Furthermore, Weber-Wulff et al. (2023) find most current popular tools exhibit a bias toward classifying outputs as human-written rather than AI-generated, and available tools perform poorly with low detection accuracy and reliability for classification. Although many studies have been successful in detecting pure machine-generated essays, there are limitations to their ability to identify AI-generated texts, particularly as AI models become increasingly sophisticated. While the detection tools are effective in many cases, the current models struggled with more complex or edited AI-generated texts (Bellini et al., 2024). Current AI-text detectors that use watermarking schemes and neural network-based methods can be easily bypassed using paraphrasing attacks meaning that the original words are replaced by random synonyms. The best detectors may only perform marginally better than random classifiers (Sadasivan et al., 2023). Krishna et al. (2024) investigates the challenges of AI-generated text detection tools often failed when the original text is paraphrased, which lower the performance of detectors. The research presents retrieval-based technique to compare the generated content to large databses of text to identify the similarity between AI-generated paraphrased content and the original source.With recent development of AI humanizers by OpenAI and other paraphrasing tools such as QuillBot, it is increasingly important to investigate the potential to improve detectors' performance when original pure AI-generated texts are modified. Chakraborty et al. (2023) find the effectiveness of detection improves significantly with increases in the number of text samples analyzed. This implies the need for further research into the relationship between dataset size and detector performance. In academic setting such as K12 schools, collecting larger sample size requires more human resources and procedures. Building on this underlying problem, the present paper explores current model performance under various versions of GenAI, such as GPT 3.5, GPT 4.0, GPT 4o, Gemini, and Gemini Advanced. Further, we investigate the impact of dataset size on detector performance and develop a novel approach in tokenization of texts when pure AI-generated texts are modified or paraphrased, for the purpose of promoting detector performance.

## 2 Materials and methods

### 2.1 Dataset

This study's human-written text and prompts are acquired from the Kaggle Automated Student Assessment Prize (ASAP) dataset sponsored by the Hewlett Foundation. We use prompt 1 as the main prompt to train and evaluate models for detection of machine-generated essays. This prompt is designed to guide high school students in writing essays on various topics. It requires students to critically think about a specific subject or problem and express their viewpoints and arguments in a short essay. ASAP prompts can cover a wide range of topics, including social issues, science, literature, history, and more, depending on the specific competition or task. Prompt 1 asks students to share their opinions about technology's effects to a local newspaper. Although not part of this research's scope, the essays are evaluated and scored by human raters from 1-6 and each text is being scored twice. The total combined score from 1-12 is record as the final score for each essay. The dataset of human-written essays used to train our models comprises 1,500 essays (Hamner et al., 2012). Research on machine-generated texts has examined various state-of-the-art GenAI models including Chat-GPT, Claude, and Gemini/Bard. This study uses prompt 1 as a question prompt with an additional requirement to generate high-scoring text based on the scoring guideline provided for 50% of the dataset, and with a specific score prompt equally distributed for the other 50%. The dataset size for each of the model versions is 500. For Chat-GPT 4o, 500 additional essays are collected to investigate the relationship between dataset size and the detectors' performance. The model versions collected in this study include Chat-GPT 3.5, 4.0, and 4o, Claude 3 and 3.5, and Gemini base and Advanced. We thus include most of the popular GenAI that are available to the public, with both base and advanced versions of each. To test our new tokenization technique, 200 essays produced by Chat-GPT 4o are paraphrased using the QuillBot paraphraser (premium mode) or Chat-GPT Humanizer. These 200 essays form a secondary dataset used to evaluate how well our new tokenization technique counters the effect of word-changing on detection of AI-generated essays. For the various stages of the experiment, the dataset is split into training, testing, and validation samples in the ratio 0.8 : 0.1 : 0.1.

### 2.2 Preprocessing

The pure machine-generated texts were collected in an excel document with their sources denoted as follows: 0 = Chat-GPT 3.5, 1 = Chat-GPT 4.0 , 2 = Chat-GPT 4o, 3 = Gemini, 4 = Gemini Advanced, 5 = Claude, 6 = Claude 3.5, 7 = Human written. The text was first normalized, including case conversion, removal of punctuation, removal of numbers, and removal of the standard headings and endings typically included in machine-generated texts. The text was then tokenized by word segmentation to split the whole text into separate words or phrases. Stop words were removed and words were reduced to their root form or base form. For the final step of text representation, we used TF-IDF for traditional machine learning algorithms and word embeddings for large language models like BERT.

TfidfVectorizer is a commonly used tool in text analysis that transforms text data into feature vectors. It helps machine learning algorithms better understand and process text data. The full name of TfidfVectorizer is Term Frequency-Inverse Document Frequency Vectorizer, which combines the concepts of term frequency and inverse document frequency. When using TfidfVectorizer, it first splits each document (e.g., an article or a piece of text) in the text data into individual words or terms. Then, TfidfVectorizer calculates the term frequency of each word in the document, which represents the frequency of that word appearing in the document. At the same time, it also calculates the inverse document frequency of each word, which represents the frequency of that word appearing in the entire text collection. By combining term frequency and inverse document frequency, TfidfVectorizer assigns a weight value to each word. This weight value indicates the importance of that word in the document and the general occurrence in the entire text collection (Kumar and Subba, 2020).

BERT (Bidirectional Encoder Representations from Transformers) embedding is a state-of-the-art word embedding technique developed by Google. Unlike traditional embeddings like Word2Vec or GloVe, which provide static word representations, BERT generates dynamic, context-aware embeddings by understanding the context of a word from both directions in a sentence. Built on the Transformer architecture, BERT uses self-attention mechanisms to weigh the importance of each word in relation to all other words in a sentence. This bidirectional approach enables BERT to capture subtle nuances and dependencies between words, providing a deeper understanding of language. BERT is pre-trained on vast amounts of text data through two main tasks: Masked Language Modeling (MLM) and Next Sentence Prediction (NSP). In MLM, random words in a sentence are masked, and BERT learns to predict these masked words using the surrounding context. NSP involves predicting whether two given sentences follow each other in the original text, helping BERT grasp inter-sentence relationships. After pre-training, BERT can be fine-tuned for specific NLP tasks such as sentiment analysis, question answering, and named entity recognition. The context-aware embeddings generated by BERT significantly enhance the performance and accuracy of these tasks, making BERT a powerful tool in the field of NLP (Devlin et al., 2018).

In addition to the typical word representation technique, we also investigate the impact of synonym replacement for AI humanizer texts on detection of AI-generated texts. To counter the effects of paraphrasing and structure changing, in addition to removing punctuation and changing words to their base forms, a new list of word synonyms is introduced before the text is tokenized by word embedding or a TF-IDF vectorizer. The synonym list is created manually by observation based on 200 essays revised by QuillBot and Chat-GPT Humanizer, identifying the most frequently-changed words by text analysis and statistical observation. We collected text data from QuillBot using the QuillBot paraphraser's premium mode and modified each word only once. Chat-GPT Humanizer automatically modifies the original text without further specifications. Each original word in the pure machine-generated text is assigned to one or more synonyms based on QuillBot and Chat-GPT Humanizer input. In model training and evaluation, the word embeddings and TF-IDF vectorizer treat the synonyms in the same way as the original word.

### 2.3 Evaluation criteria

We use five different evaluation scores to measure the performance of each model, including Precision, Recall, F1, Accuracy, and Quadratic Weighted Kappa (QWK) scores. In the measured matrix, TP (True Positive) denotes the correct texts that are identified as machine-generated. TN (True Negative) denotes the correct texts that are identified as human-written. FP (False Positive) means the texts that are human-written being misclassified as machine-generated. FN (False Negative) means the texts that are machine-generated being misclassified as human-written. Precision, Recall, F1, and Accuracy scores are be defined as follows:


(1) $$\begin{array}{l} \text{Precision}=\frac{TP}{TP+FP} \end{array}$$

(2) $$\begin{array}{l} \text{Recall}=\frac{TP}{TP+FN} \end{array}$$

(3) $$\begin{array}{l} \text{F1}=2\cdot \frac{\text{Precision}\cdot \text{Recall}}{\text{Precision}+\text{Recall}} \end{array}$$

(4) $$\begin{array}{l} \text{Accuracy}=\frac{TP+TN}{TP+TN+FP+FN} \end{array}$$

Precision measures the accuracy of a model's positive predictions; specifically, it is the ratio of true positive predictions to the total number of positive predictions. Recall, or sensitivity, quantifies the model's ability to identify positive instances, calculated as the ratio of true positive predictions to the total number of actual positive instances. The F1 score is the harmonic mean of Precision and Recall, providing a balanced metric that considers both the precision and recall of the model. Accuracy assesses the overall correctness of the model's predictions, calculated as the ratio of correctly predicted instances (both positive and negative) to the total number of instances. These metrics collectively provide a comprehensive evaluation of a model's performance, highlighting different aspects of its predictive capabilities. The QWK score is defined as follows:


(5) $$\begin{array}{l} \kappa =1-\frac{\sum_{i,j}w_{i,j}O_{i,j}}{\sum_{i,j}w_{i,j}E_{i,j}} \end{array}$$

where *w*_*i, j*_ denotes the quadratic weights, *O*_*i, j*_ is the observed frequency, and *E*_*i, j*_ is the expected frequency.

In essence, our study becomes a binary classification problem in identifying the source of texts that are either machine-generated or human-written. Unlike simple accuracy, QWK accounts for the severity of different types of incorrect predictions by applying quadratic weights, making it particularly useful for assessing model performance in scenarios where the distinction between correct and incorrect classifications is important. QWK scores range from -1 (complete disagreement) to 1 (perfect agreement), and a score of 0 indicates agreement no better than chance, thereby providing a more nuanced measure of model reliability and consistency (Doewes et al., 2023).

### 2.4 Models

To evaluate the detection of AI-generated texts and AI-humanized texts, we utilize two different categories of models: classical machine learning models and large language models. Among classical machine learning models, we select SVMs, logistic regression, and extreme gradient boosting (XGBoost). SVM is a powerful classification algorithm that works by finding the optimal hyperplane that maximizes the margin between different classes, making it highly effective for high-dimensional data (Suthaharan and Suthaharan, 2016). Logistic regression is a widely-used statistical model for binary classification that estimates the probability of a class label based on a logistic function. This type of model is known for its simplicity and interpretability (LaValley, 2008). XGBoost, an advanced implementation of gradient boosting, is designed for speed and performance, and constructs an ensemble of decision trees by sequentially adding models that correct the errors of previous models, thereby improving accuracy and reducing overfitting (Chen and Guestrin, 2016). These models each have unique strengths, making them suitable for a variety of machine learning tasks, including the detection of AI-generated and AI-humanized texts.

We employ large language models such as BERT, Electra, and RoBERTa to evaluate the detection of AI-generated texts. BERT (Bidirectional Encoder Representations from Transformers) is a powerful pre-trained model that understands the context of text by training bidirectionally across all layers, making it highly effective for a wide range of natural language processing tasks (Devlin et al., 2018). Electra (Efficiently Learning an Encoder that Classifies Token Replacements Accurately) employs a novel pre-training strategy that improves training efficiency and accuracy by having the model learn to detect whether a word in the input has been replaced, instead of the traditional generate-and-discriminate approach (Clark et al., 2020). RoBERTa (A Robustly Optimized BERT Pretraining Approach) builds on BERT by optimizing the pre-training process with larger datasets and longer training times, enhancing performance and robustness (Liu et al., 2019). These large language models, with their advanced capabilities in understanding and generating natural language text, are used to detect AI-generated and AI-humanized texts in this study. GPT-Zero is an advanced AI model designed for detecting AI-generated texts and distinguishing them from human-written content. We use it as an example of an AI-checking tool that is available online and is current popular, and contrast its performance with other algorithms in identifying AI-generated text that has been paraphrased (Chaka, 2023).

## 3 Results

Detailed performance data for our six models are provided in the supplementary material. The best performing model in terms of highest precision score is highlighted in each table. Overall, our results show that support vector machine (SVM) models outperform both XGBoost and logistic regression in the detection of AI-generated texts. Specifically, in classic machine learning models, SVM achieved the highest Quadratic Weighted Kappa (QWK) score of 0.972 when detecting Gemini base model texts in Table 1. This indicates that the SVM model is highly effective in distinguishing between human-written and machine-generated content across various GenAI models. Moreover, the SVM's QWK scores remained consistently high across all categories of texts, with an average score of around 0.95, further demonstrating its robustness in handling diverse text inputs.

**Table 1:** Evaluation matrix of support vector machine model.

**GenAI type**	**Precision**	**Recall**	**F1**	**Accuracy**	**QWK**
GPT 3.5	0.931	0.959	0.944	0.973	0.963
GPT 4.0	0.923	0.902	0.911	0.955	0.948
GPT 4o	0.924	0.939	0.931	0.966	0.956
Gemini	0.940	0.940	0.940	0.970	0.972
Gemini Advanced	0.900	0.918	0.909	0.955	0.962
Claude	0.920	0.939	0.929	0.965	0.957
Claude 3.5	0.916	0.920	0.918	0.959	0.943

The highlighted row indicates the highest precision score observed among the detectors.

The superior performance of SVM can be attributed to its ability to handle high-dimensional data, which is crucial when dealing with text classification tasks where features such as linguistic patterns, syntactic structures, and vectorized word representations are numerous. Additionally, SVM's regularization techniques help prevent overfitting, ensuring it generalizes well to different samples, even when the AI-generated texts exhibit variations. Its capacity to capture complex relationships between data points through non-linear decision boundaries enhances its ability to detect nuances to differentiate human-written texts from AI-generated content.

Logistic regression and XGBoost also performed well, with both models achieving high QWK scores of 0.961 and 0.964, respectively, in the detection of Gemini base model texts in Tables 2, 3. Despite their differences in algorithmic approaches, XGBoost which is a tree-based classifier and logistic regression, a linear model, their similar performance in terms of QWK score highlights that both models reveals sufficient performance for the text classification. The findings from the experiment confirm that traditional machine learning models, which are easier to interpret and require fewer computational resources than neural models, still perform exceptionally well in AI text detection. All three models consistently maintained accuracy rates above 90%, which clearly illustrates that human-written and machine-generated texts can be effectively distinguished using these models.

**Table 2:** Evaluation matrix of logistic regression model.

**GenAI type**	**Precision**	**Recall**	**F1**	**Accuracy**	**QWK**
GPT 3.5	0.896	0.865	0.880	0.939	0.928
GPT 4.0	0.864	0.878	0.871	0.936	0.932
GPT 4o	0.884	0.867	0.875	0.937	0.928
Claude	0.908	0.912	0.910	0.955	0.944
Gemini	0.904	0.900	0.902	0.951	0.961
Gemini Advanced	0.886	0.902	0.894	0.948	0.932
Claude 3.5	0.884	0.898	0.891	0.946	0.948

The highlighted row indicates the highest precision score observed among the detectors.

**Table 3:** Evaluation matrix of XGBoost model.

**GenAI type**	**Precision**	**Recall**	**F1**	**Accuracy**	**QWK**
GPT 3.5	0.906	0.904	0.905	0.958	0.948
GPT 4.0	0.916	0.851	0.882	0.939	0.928
GPT 4o	0.898	0.865	0.881	0.940	0.934
Gemini	0.934	0.921	0.928	0.964	0.964
Gemini Advanced	0.918	0.929	0.924	0.962	0.948
Claude	0.896	0.877	0.886	0.943	0.937
Claude 3.5	0.878	0.861	0.869	0.934	0.941

The highlighted row indicates the highest precision score observed among the detectors.

Among the large language models tested (BERT, Electra, and RoBERTa), BERT in Table 4 underperformed compared to Electra in Table 5 and RoBERTa in Table 6. BERT's lower QWK score suggests that it may struggle with capturing the subtle linguistic patterns that distinguish human from machine-generated content. However, one advantage BERT had was its lower computational cost–it required less time and fewer resources during training, which could be advantageous in contexts where processing power and time is limited.

**Table 4:** Evaluation matrix of BERT model.

**GenAI type**	**Precision**	**Recall**	**F1**	**Accuracy**	**QWK**
GPT 3.5	0.904	0.866	0.885	0.941	0.945
GPT 4.0	0.914	0.882	0.898	0.944	0.938
GPT 4o	0.862	0.857	0.859	0.930	0.912
Gemini	0.908	0.901	0.904	0.952	0.946
Gemini Advanced	0.896	0.885	0.891	0.945	0.935
Claude	0.902	0.886	0.894	0.947	0.936
Claude 3.5	0.896	0.905	0.901	0.951	0.954

The highlighted row indicates the highest precision score observed among the detectors.

**Table 5:** Evaluation matrix of Electra model.

**GenAI type**	**Precision**	**Recall**	**F1**	**Accuracy**	**QWK**
GPT 3.5	0.924	0.920	0.922	0.961	0.957
GPT 4.0	0.916	0.914	0.915	0.958	0.943
GPT 4o	0.919	0.900	0.909	0.954	0.961
Gemini	0.923	0.913	0.918	0.959	0.957
Claude 3.5	0.944	0.922	0.933	0.966	0.959
Gemini Advanced	0.924	0.920	0.922	0.961	0.959
Claude	0.918	0.924	0.921	0.961	0.953

The highlighted row indicates the highest precision score observed among the detectors.

**Table 6:** Evaluation matrix of RoBERTa model.

**GenAI type**	**Precision**	**Recall**	**F1**	**Accuracy**	**QWK**
GPT 3.5	0.916	0.902	0.909	0.954	0.945
GPT 4.0	0.898	0.865	0.881	0.940	0.936
GPT 4o	0.899	0.862	0.880	0.939	0.921
Gemini	0.906	0.919	0.912	0.957	0.961
Claude	0.906	0.942	0.924	0.963	0.962
Gemini Advanced	0.900	0.882	0.891	0.945	0.941
Claude 3.5	0.904	0.850	0.876	0.936	0.928

The highlighted row indicates the highest precision score observed among the detectors.

Electra, on the other hand, outperformed BERT, achieving a QWK score of 0.961 in detecting GPT 4o-generated texts. Electra's performance is largely due to its unique pre-training process. It learns to predict whether a token has been replaced, unlike the masked language model (MLM) approach used by BERT. This allows Electra to detect subtle anomalies or unnatural token replacements that may be more common in AI-generated texts (Clark et al., 2020). Additionally, Electra is more resource-efficient compared to models like BERT, enabling it to process more tokens during training and develop better contextual representations. RoBERTa achieved the highest QWK score among the LLMs, with a score of 0.962 when detecting texts from the Claude base model. Interestingly, despite claims by developers that more advanced versions of GenAI models are more sophisticated, our detection algorithms were still able to accurately detect these “more powerful” models due to their persistent patterns in language structure and word selection preferences.

To better understand the relationship between dataset size and model performance, we conducted additional tests using an expanded dataset that included 1,000 GPT 4o-generated texts. The SVM and Electra models were selected for this experiment, and we evaluated their performance on dataset sizes ranging from 100 to 1,000 texts, increasing by increments of 100. The results, shown in Figure 2, indicate a clear positive correlation between dataset size and model performance, particularly for smaller datasets. However, as the dataset size reached 300 texts, the rate of performance improvement began to fluctuate minimally. This suggests that while larger datasets can help improve model performance, there may be diminishing returns beyond a certain dataset size.

SVM's ability to handle large and high-dimensional datasets contributes to its stable performance as dataset size increases. Similarly, Electra's discriminative training approach allows it to generalize effectively, even with larger datasets. Both models showcase strong adaptability to various dataset sizes, but their peak performance is achieved once a sufficient volume of data is provided.

As discussed in the introduction, one key challenge in detecting AI-generated content is the use of paraphrasing tools, which helps to evade detection. To explore this, we tested two popular paraphrasing tools–GPT-Humanizer (an AI-based tool integrated within the OpenAI platform) and QuillBot (a widely used online paraphraser). We selected 200 GPT 4o-generated essays and paraphrased them using these tools. We then evaluated the impact of these paraphrasing tools on detection performance using SVM and Electra models, both in their original (unmodified) and modified states (after incorporating synonym replacement techniques).

**Figure 1:**
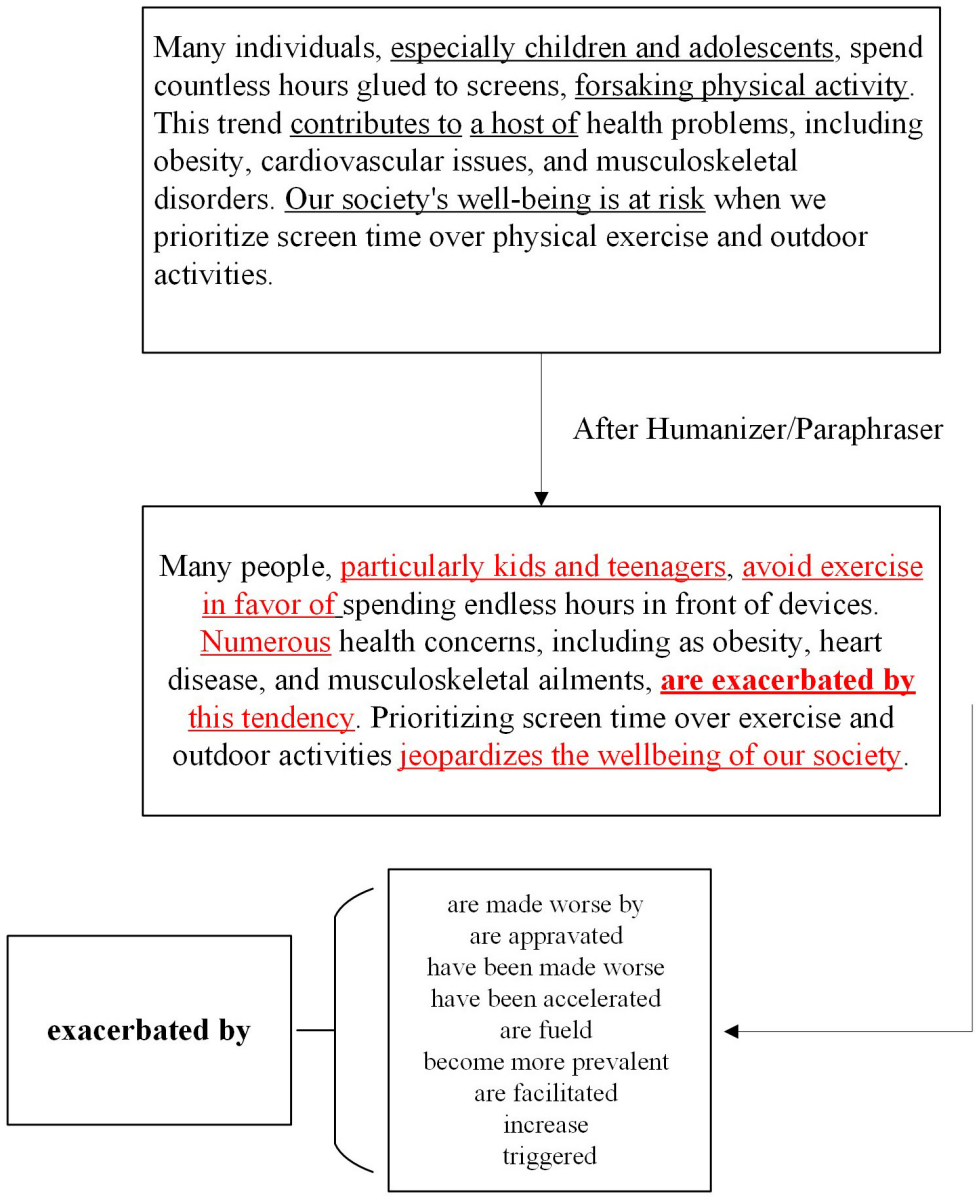
Word replacement.

The results, presented in Table 7, demonstrate that paraphrasing significantly decreases the effectiveness of traditional AI-detection models. However, by using a synonym replacement technique, we were able to counteract this effect, increasing the detection rate by approximately 30 percentage points. This technique involves identifying red-flagged words that were altered by the paraphrasing tool and replacing them with synonyms that match the original word's meaning as showed in Figure 1. For example, in one instance, the phrase “fervent advocate” was paraphrased into several alternative expressions. By tracking and reversing these changes, we were able to improve the detection accuracy significantly.

**Table 7:** Impact of synonym replacement methods on model accuracy scores.

**Modification method**	**SVM**	**Electra**	**SVM (modified)**	**Electra (modified)**	**GPT-zero**
QuillBot	0.665	0.645	0.961	0.950	0.04
GPT-Humanizer	0.62	0.625	0.955	0.917	0.16

In comparison, GPT-Zero, a popular AI-content checker, struggled to detect paraphrased content. The benchmark classification for AI content is set at 50%, meaning that if GPT-Zero identifies content as being more than 50% AI-generated, it tags it as machine-written. However, after paraphrasing, GPT-Zero's performance dropped, particularly when paraphrasing tools replaced sophisticated words like “ecstatic” with simpler alternatives like “happy.” Additionally, changes in sentence structure (e.g., splitting complex sentences into simpler, shorter ones) further hindered GPT-Zero's detection capability, as seen when long compound sentences such as “not only ... but also” were paraphrased into two separate, simpler sentences. This exposes a significant weakness in GPT-Zero when dealing with paraphrased content.

Our study highlights the robustness of SVM and Electra models in detecting AI-generated texts, even with large dataset sizes. The high performance for both modelscan be attributed to their ability to handle high-dimensional data, detect subtle anomalies in text, and effectively generalize across diverse samples. While paraphrasing presents a challenge, our synonym replacement technique proves highly effective in mitigating this issue. Among the large language models, RoBERTa shows the highest performance in AI detection, though BERT remains a viable option for resource-constrained environments. In real world scenarios, the selection of specific models also requires balancing between time and resources required. Tools like GPT-Zero, although popular, may require significant improvements to handle paraphrased content effectively. Overall, our findings emphasize the need for continuous advancements in detection algorithms to keep pace with evolving AI-generation techniques.

## 4 Discussion

Most previous tests of GenAI models use only a single version of each model. We compare different models' performance including base versions and latest, most advanced, versions. Although the models can produce high quality texts, there are detectable differences between human-written and machine-generated texts even for the latest versions. Furthermore, the most advanced versions of the models are not necessarily harder to detect than base versions. Various detection models are trained to evaluate the difference between human-written and machine-generated essays and all achieve more than 90% accuracy, illustrating that using current machine learning and large language models, detection of pure original AI essays is relatively easy.

In addition to analyzing current GenAI models, we also propose a novel approach to analyzing texts. Students commonly use paraphrasing tools and checking tools such as GPT-Zero to avoid being caught using AI to cheat on academic work.Therefore we use synonyms to counter the effect of word changing in detecting AI-generated essays. In our prompt 1 setting, we find this modification method to be effective; it enables our detection models to better analyze and classify texts' sources without being fooled by paraphrasing. GPT-Zero, one of the most popular online checking tools, is less effective when paraphrasing is extensively used. In the setting for academic integrity, the experiment results indicates the need of continous development of detection tools to counter the advancement of AI humanizers and improve the detection performance.

**Figure 2:**
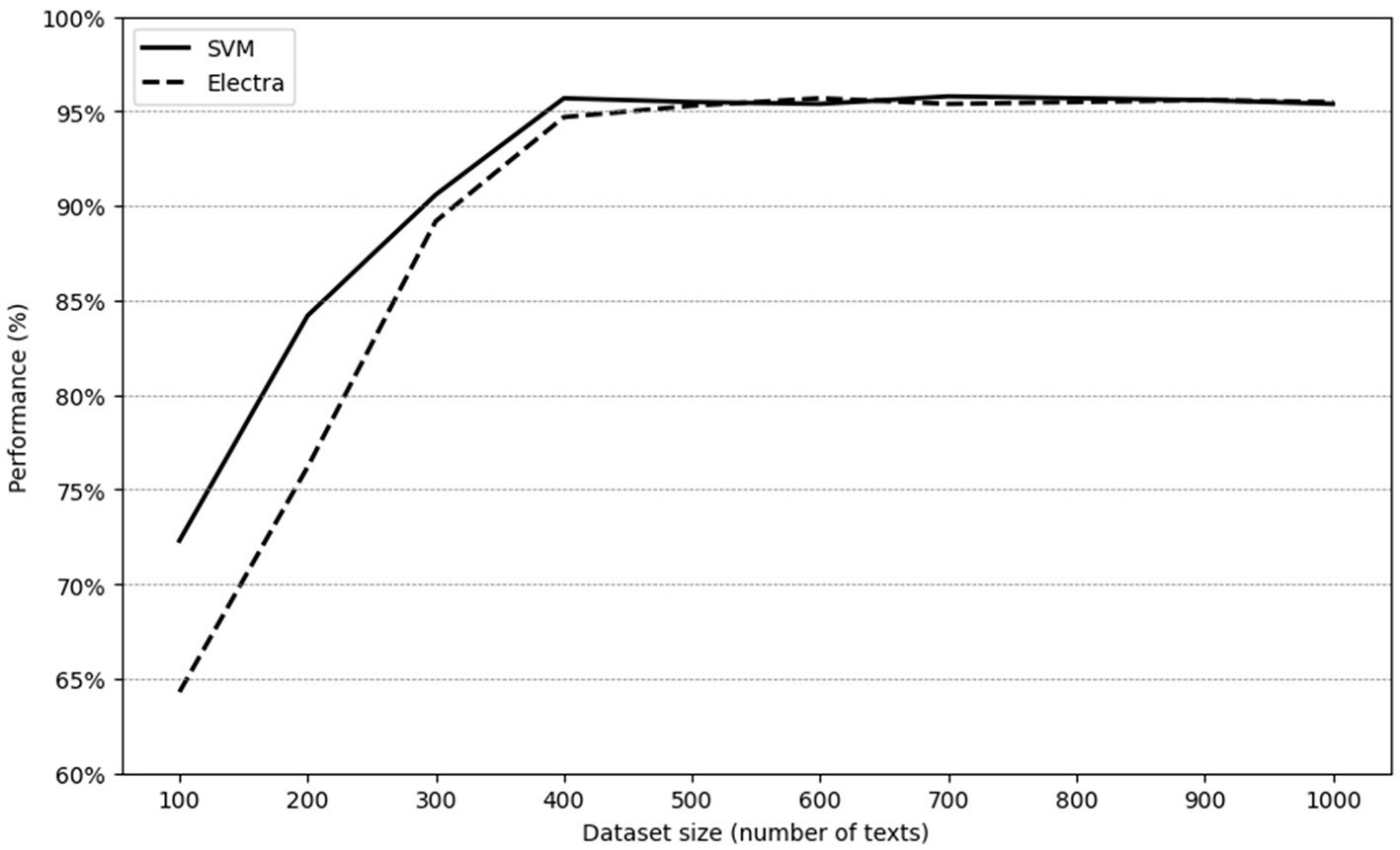
Model performance in comparison with dataset size.

This study also examines the relationship between dataset size and model performance. When investigating detection of GenAI essays, researchers often need to collect large numbers of essays and carefully examine their content to avoid repetitive texts and better simulate real-world scenarios. Our results in Figure 2 inform the data collection process, indicating the number of texts needed to conduct effective research.

However, this study has limitations. The research focuses on a single prompt, and our synonym replacement technique was applied specifically to this context. This tailored approach may inflate the performance of our detection models, raising questions about the technique's generalizability across different prompts and academic tasks. Larger datasets in the future experiment is needed to validate the results and expand the study.

Future research should explore how various paraphrasing methods impact detection performance, especially in academic settings where the stakes are high for preventing cheating. Detection models must reach near-100% accuracy and recall to avoid misclassifying students or failing to detect cheating. Moreover, while this study focuses on academic integrity, the principles of detecting humanized AI-generated content have broader implications in fields such as advertising and spam email detection. Developing more robust AI-detection tools will benefit not just academia, but also other sectors where AI-generated content is prevalent.

## Data Availability

The raw data supporting the conclusions of this article will be made available by the authors, without undue reservation.
